# Differentiating the structure of PtNi octahedral nanoparticles through combined ADF–EDX simulations

**DOI:** 10.1186/s40679-018-0053-x

**Published:** 2018-02-20

**Authors:** Katherine E. MacArthur, Marc Heggen, Rafal E. Dunin-Borkowski

**Affiliations:** Ernst Ruska-Centre for Microscopy and Spectroscopy with Electrons and Peter Grünberg Institute, Forschungszentrum Jülich, 52425 Jülich, Germany

**Keywords:** STEM, EDX, Nanoparticles, PtNi, Composition, Simulation, Octahedra

## Abstract

Advances in catalysis rely on the synthesis and characterisation of nanoparticles that have tailored structures and compositions. Although energy-dispersive X-ray (EDX) spectroscopy can be used to study local variations in the compositions of individual supported nanoparticles on the atomic-scale in the scanning transmission electron microscope, electron beam induced damage and contamination can preclude the use of long exposure times and tomographic approaches. Here, we perform simulations of EDX maps of seven different octahedral PtNi nanoparticles for a selection of crystallographic orientations and tilts, to evaluate which of them can be distinguished from elemental mapping performed in only one orientation.

**Electronic supplementary material:**

The online version of this article (10.1186/s40679-018-0053-x) contains supplementary material, which is available to authorized users.

## Background

Pt-based bimetallic nanoparticles have demonstrated great promise as catalysts for the oxygen reduction reaction (ORR) in hydrogen fuel cells [1]. The addition of a second transition metal, such as Ni or Co, not only reduces the cost of the catalyst due to the reduction in Pt metal loading, but also provides an increase in activity. Recently, octahedral PtNi nanoparticles have attracted considerable interest, as it was reported that the Pt_3_Ni (111) alloy surface has an exceptionally high activity for the ORR [2]. Its activity is 10 times higher than that of the Pt (111) surface and 90 times higher than that of state-of-the-art Pt/C catalysts. Therefore, there is great benefit to understanding the structure and composition of the near-surface layers on such nanoparticles. Intriguingly, their compositions have been reported to oscillate from one layer to the next, with Pt-rich outermost and third layers and a Ni-rich second layer. The outermost Pt-rich atomic layer is often referred to as a Pt skin [3–5]. In light of the exceptionally high ORR activity demonstrated by Pt_3_Ni (111) surfaces, large efforts have been dedicated to the synthesis and characterisation of uniform-Pt-skin octahedral Pt-Ni nanoparticles [6–10]. A strong link between the atomic-scale structures of such octahedral nanoparticles and their catalytic performance has been demonstrated in a number of studies. Cui et al. [11] described inhomogeneous compositional distributions in shaped Pt alloy nanoparticles, in the form of Pt-rich frames and Ni-rich facets. They showed that the selective etching of Ni-rich {111} facets during electrochemical cycling results in the formation of concave octahedra, thereby exposing less active facets and leading to a significant reduction in activity. Subsequently, it was shown that anisotropic growth is responsible for such inhomogeneous compositional distributions [12], with rapid growth of Pt-rich hexapods/concave octahedra along ⟨100⟩ directions preceding the deposition of a Ni-rich phase in the concave {111} regions. Different structural modifications contain Pt and Ni distributions beyond the basic “Pt hexapod” morphology. For instance, in 2015 Oh et al. investigated CO-induced compositional segregation in Ni-rich octahedral Pt-alloy nanoparticles [13]. They reported the formation of Ni octahedra encased by octahedral Pt frameworks, with three intersecting perpendicular Pt axes. Growth was demonstrated to start with a Pt-rich core, which transformed into a slightly concave octahedron, followed by the formation of an octahedral core–shell Pt@Ni nanoparticle. The protruding ends of the Pt-rich concave nanoparticle continued to form during nanoparticle growth by selectively recruiting Pt over Ni precursors. A final heat treatment under CO led to Pt migration from the core to the surface, resulting in the formation of Pt-rich lines along the ⟨110⟩ edges and the three perpendicular inner axes. Although previous work has reported a variety of possible models for octahedral PtNi nanoparticles, an exact atomic-scale understanding of their structure in three dimensions (3D) is highly challenging to obtain experimentally. For example, the presence of a Pt-rich skin [7] has yet to be confirmed unequivocally, emphasising the importance of performing precise atomic-scale investigation of such structures.

The scanning transmission electron microscope (STEM) is an invaluable tool for the structural and compositional analysis of bimetallic nanoparticles. The high-angle annular dark-field (ADF) signal provides information about local variations in specimen composition and thickness in the form of so-called ‘Z-contrast’. Several researchers have quantified the absolute intensity of this signal, to determine sample thickness (of single-element samples) [14–17] and composition (when the sample thickness is well known) [18, 19]. When the sample composition and thickness are both unknown, as is the case with the PtNi octahedra that we describe below, the ADF STEM signal alone provides insufficient information to characterise the particles fully. Local elemental characterisation is possible using energy dispersive X-ray (EDX) spectroscopy or electrons energy-loss spectroscopy (EELS), which allow the measurement of variations in composition within a single nanoparticle in the form of elemental maps. Of these two techniques, EDX is preferable for PtNi nanoparticles, in large part, due to the high energy-loss of the Pt edge. Unfortunately, the interpretation of such maps is complicated by the fact that they are two-dimensional representations of 3D structures. One way to resolve the 3D problem is through tomography. Atomic resolution tomography has been used to determine the 3D structures of nanoparticles [20–22], however, not yet with direct (spectroscopic) compositional information. Although EDX tomography [22–25] would provide the required 3D compositional information, many nanoparticle structures are not able to survive the high electron dose that is required for such investigations. As one of the fundamental principles of tomography is that each projection is recorded from the same structure, electron beam induced damage or contamination can limit the accuracy of such measurements. Time is also a factor, as imaging nanoparticles in only one projection is often the only recourse during in situ experiments, as well as making it easier to study more nanoparticles during a TEM session and providing faster feedback to adjust synthesis processes. Here, we assess the feasibility of using an alternative approach to tomography by the direct comparison of EDX maps of structurally and compositionally symmetrical PtNi nanoparticles with image simulations, similar to the approach that is often used for the interpretation of high-resolution TEM images of nanoparticles [26, 27]. We present simulated atomically-resolved ADF STEM images and EDX maps of seven model structures and discuss which of them are distinguishable from each other. Such results are intended to assist in careful experimental design for the accurate structural distinction of real nanoparticles.

## Methods

**Fig. 1:**
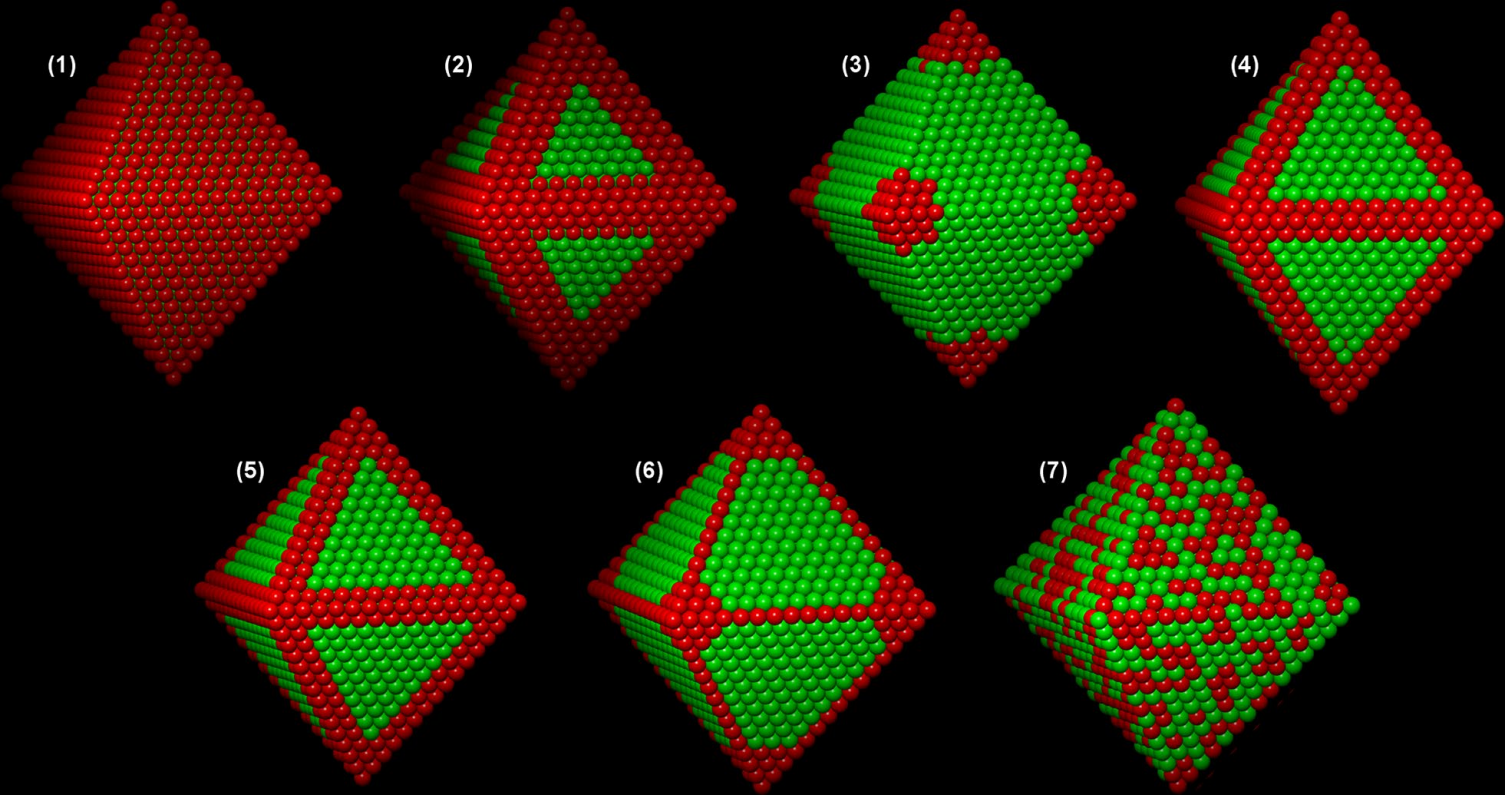
Three-dimensional renderings of the seven investigated nanoparticle structures, with Pt atoms shown in red and Ni atoms shown in green: (1) Pt monolayer over a Ni core; (2) Pt edges over a Ni core; (3) Pt hexapod; (4) Pt hexapod and edges; (5) Pt hexapod, Pt edges and Pt core [29]; (6) Ni facets that are not connected to each other; (7) random alloy structure

**Fig. 2:**
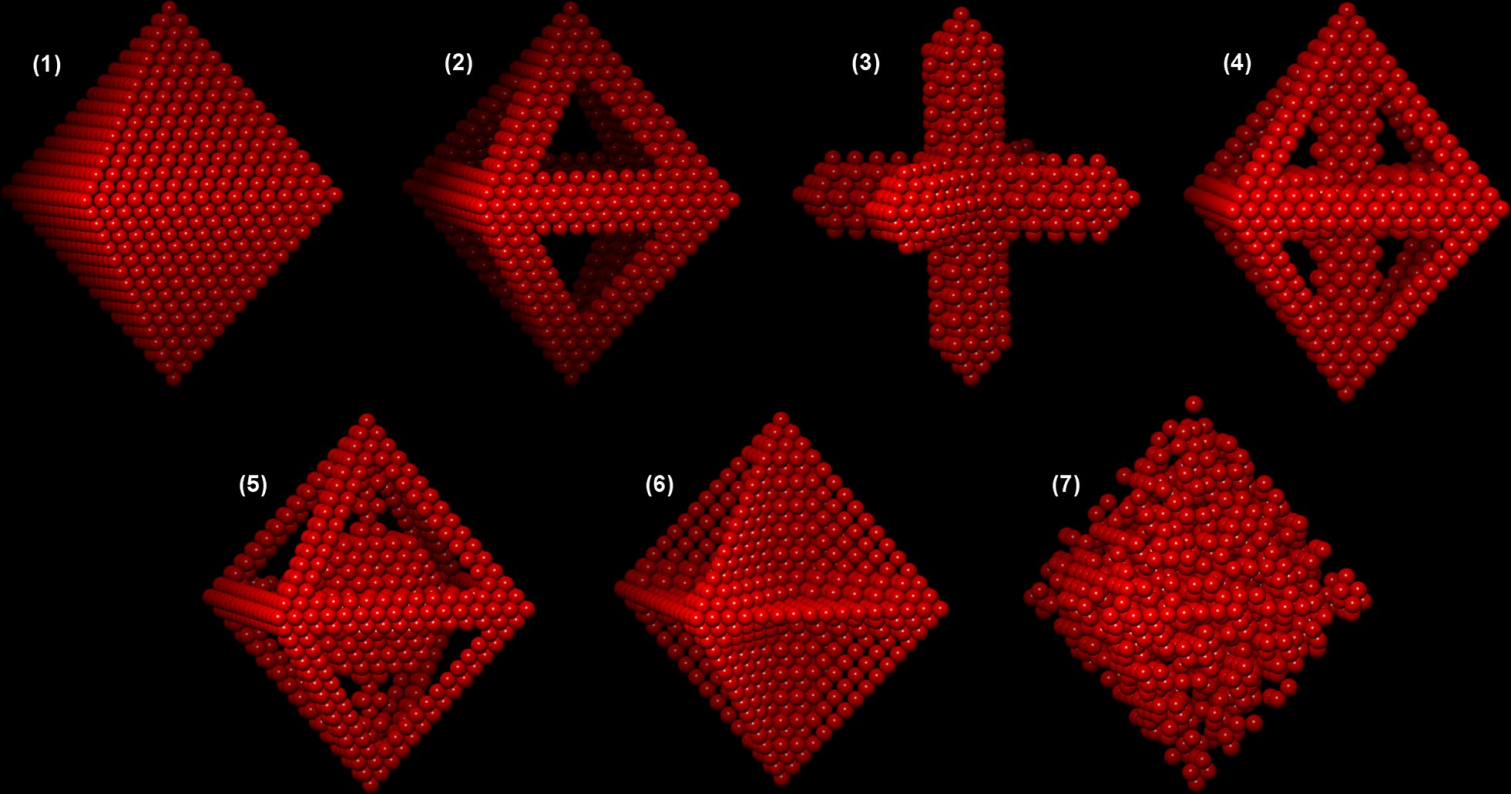
Three-dimensional renderings of the seven investigated nanoparticle structures, with only the Pt atoms shown: (1) Pt monolayer over a Ni core; (2) Pt edges over a Ni core; (3) Pt hexapod; (4) Pt hexapod and edges; (5) Pt hexapod, Pt edges and Pt core [29]; (6) Ni facets that are not connected to each other; (7) random alloy structure

The model structures, which are summarised in Table 1, are referred to here as: (1) Pt shell, (2) Pt edges, (3) Pt hexapod, (4) Pt hexapod and edges, (5) Pt hexapod, edges and core, (6) Ni facets and (7) alloy. Similar model structures have been proposed in the literature based on qualitative comparisons of experimental results with STEM images and/or EDX maps. For example, structure (5) is similar to that proposed by Oh et al. [13], while structures (3) and (6) were described by Cui et al. and Gan et al. [11, 28]. However, they have yet to be validated using electron tomography or using comparisons with simulated STEM images and/or EDX maps. The compositions of the model structures are kept as close to 40 at% Pt as possible, so that details in the simulated STEM images and EDX maps can be compared with each other without needing to consider differences in their average composition. 3D renderings of each structure are shown in Figs. 1 and 2, with Pt atoms shown in red and Ni atoms shown in green. In each case, we assume here that the nanoparticle composition follows the symmetry of the particle shape. This is often a reasonable approximation experimentally, in particular when there is nothing in the synthesis method that affects the crystalline symmetry of the structure. The influence of variations in composition between the different segments of a nanoparticle is not considered in the present study.

**Table 1:** Summary of the investigated structures

Name	Description	Number of Pt atoms	Number of Ni atoms	Composition
(1) Pt shell	Pt monolayer shell with Ni core	902	1834	32.97% Pt
(2) Pt edges	Pt only at nanoparticle edges	1074	1662	39.25% Pt
(3) Pt hexapod	Pt seed crystal grown out in ⟨100⟩ directions to form a hexapod structure	1040	1696	38.01% Pt
(4) Pt hexapod and edges	As above, but with additional Pt decoration on the edges of the nanoparticle	1118	1618	40.86% Pt
(5) Pt hexapod, edges and core	As above, but with a Pt cuboctahedron in the core	1064	1672	38.89% Pt
(6) Ni facets	Ni concentrated at the centre of each facet and unconnected in the nanoparticle	1136	1600	41.52% Pt
(7) Alloy	Random mixture of Pt and Ni atoms	1094	1642	39.99% Pt

For each structure, ADF STEM images and EDX maps for the Pt-L and Ni-K lines were simulated using the μSTEM code developed in the University of Melbourne [30, 31]. This code has previously been used to achieve an exact match between experimental and simulated X-ray counts on an absolute scale, requiring a careful calibration of experimental parameters [32]. The present simulations were carried out for an accelerating voltage of 200 kV using an aberration corrected probe with a convergence semi-angle of 25 mrad. The ADF detector collection semi-angles were 75–180 mrad, the X-ray signals were simulated assuming a full 4*π* sr collection solid angle, and the total number of X-rays was integrated over all possible sub-shells for the Ni-K and Pt-L lines. Specimen tilt was implemented by applying a matrix rotation to the input structure file and adjusting the slicing in the *z* direction to ensure that each atomic potential was allocated to one slice. In this preliminary investigation, the lattice parameter of each structure was kept constant at the value for bulk Pt of 0.3924 nm. In reality, such particles are likely to be strained as a result of differences in the atomic sizes of Pt and Ni. However, such strain distributions can only be incorporated realistically in simulations of electron propagation once the composition of the sample is known.

To establish whether or not variations between the different nanoparticle structures are detectable above a realistic noise level, X-ray counts were simulated on an absolute scale according to the equation [33]

$$N = I_{\text{inc}} \tau F_{\text{ion}} \left( {t,X_{\text{abs}} } \right)\omega \left( {\frac{\varOmega }{4\pi }} \right)D_{\text{eff}}$$

where *N* is the number of X-ray counts. *I*_inc_ is the incident beam current (a typical probe current for atomic resolution mapping at 200 kV on an aberration corrected Titan is 40 pA, which corresponds to $2.5 \times 10^{8} \;{\text{e}}/{\text{s}}$). $\tau$ is the dwell time (for a 110 × 110 pixel map recorded over 10 min, this corresponds to 24.8 ms/pixel. $F_{\text{ion}} \left( {t,X_{\text{abs}} } \right)$ is the output of the μSTEM code (defined as the fraction of the incident electrons that result in ionization, incorporating a depth-dependent absorption correction), $\omega$ is the fluorescence yield (here, we used 0.412 for the Ni K shell and 0.325 for the Pt L shell [34]), $\varOmega$ is the solid angle of the EDX detector (for the Super-X quadrant detector this is typically quoted as 0.7 srad) and *D*_eff_ is the detector efficiency (assumed to be 1, as silicon drift detectors have an efficiency close to 1 in the energy range of X-rays being investigated here [35]). Once the images were determined in absolute counts, Poisson noise was added (as the standard deviation equal to the square root of the number of counts), as this is accepted to be the largest and most limiting source of error in TEM–EDX analysis. All line profiles are presented below as absolute counts, assuming a dwell time of 1 s per point. Other dwell times are specified where necessary. All images within one compound image are represented on the same intensity scale.

## Results and discussion

**Fig. 3:**
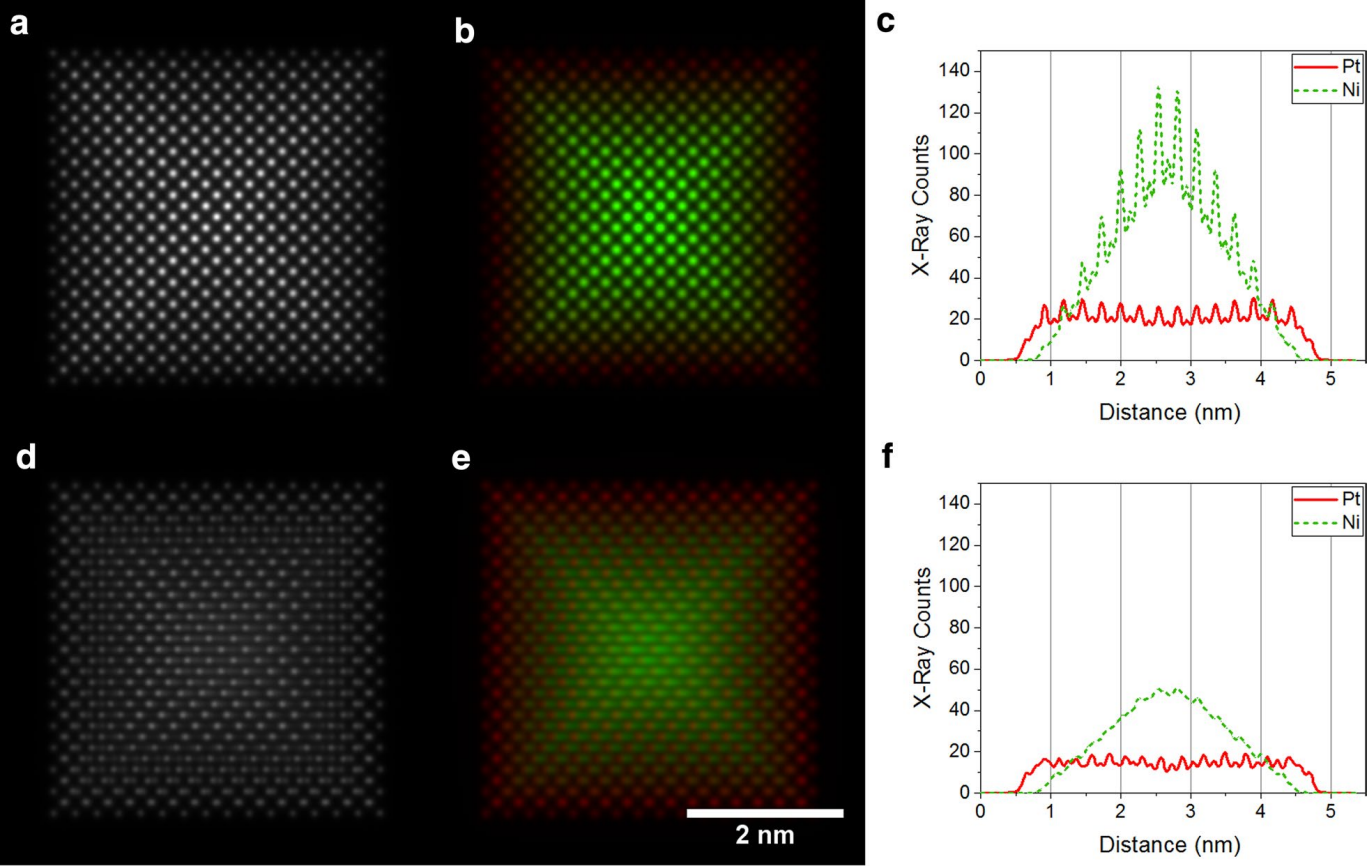
Simulated ADF STEM images and corresponding EDX maps of Pt-shell nanoparticle structure (1), viewed **a**, **b** along a ⟨100⟩ zone axis and **d**, **e** tilted by 5° from the ⟨100⟩ zone axis towards the ⟨110⟩ zone axis. The intensity in **e** has been doubled to make the figure visible on the same intensity scale as the on-axis map (**b**). Corresponding line profiles taken horizontally across the centre of the nanoparticle are shown in **c** and **f**, assuming a dwell time of 1 s per point and a pixel width of 11 pm. The Pt-L signal is shown in red, while the Ni-K signal is shown in green

Figure 3a, and b show a simulated ADF STEM image and a simulated EDX map of Pt monolayer structure (1), respectively, viewed down the ⟨100⟩ zone-axis (i.e. from the vertex of the nanoparticle). Interestingly, the outer edges of the nanoparticle are not visibly brighter in the ADF STEM image, as the Pt shell is very thin in the electron beam direction in this projection. Without accurate quantification, the figures are challenging to interpret and one could easily infer a lower Pt content than the true value of 33%. After tilting the particle by only 5° towards the ⟨110⟩ zone axis, the core–shell structure becomes much clearer in the EDX map (Fig. 3e). This difference arises in part due to electron channelling, which can be suppressed by tilting the nanoparticle away from a zone axis orientation [36, 37]. The term ‘electron channelling’ describes the action of an aligned column of atoms as a set of miniature lenses, which results in an additional focusing effect on the electron beam. The second atom in a column then contributes more to the image intensity than the first and so on. Along a longer column, the beam oscillates in intensity in a manner similar to a standing wave, resulting in the signal intensity not being proportional to the number of atoms in the column [37, 38]. For small sample tilt angles away from the zone axis orientation, the EDX map reflects the projected thickness of each element more closely, making it simpler to interpret. A tilt angle of 5° is normally sufficient [37, 38] to suppress channelling. The effect of electron channelling can also be seen in Additional file 1: Figures S1(5). Although structure (5) has a truncated octahedral nanoparticle at its centre, the simulated image suggests the presence of two Ni-rich columns in the nanoparticle. After tilting by 5°, these excessively bright Ni columns are no longer present, indicating that they are an artefact of electron channelling (see Fig. 5(5)).

**Fig. 4:**
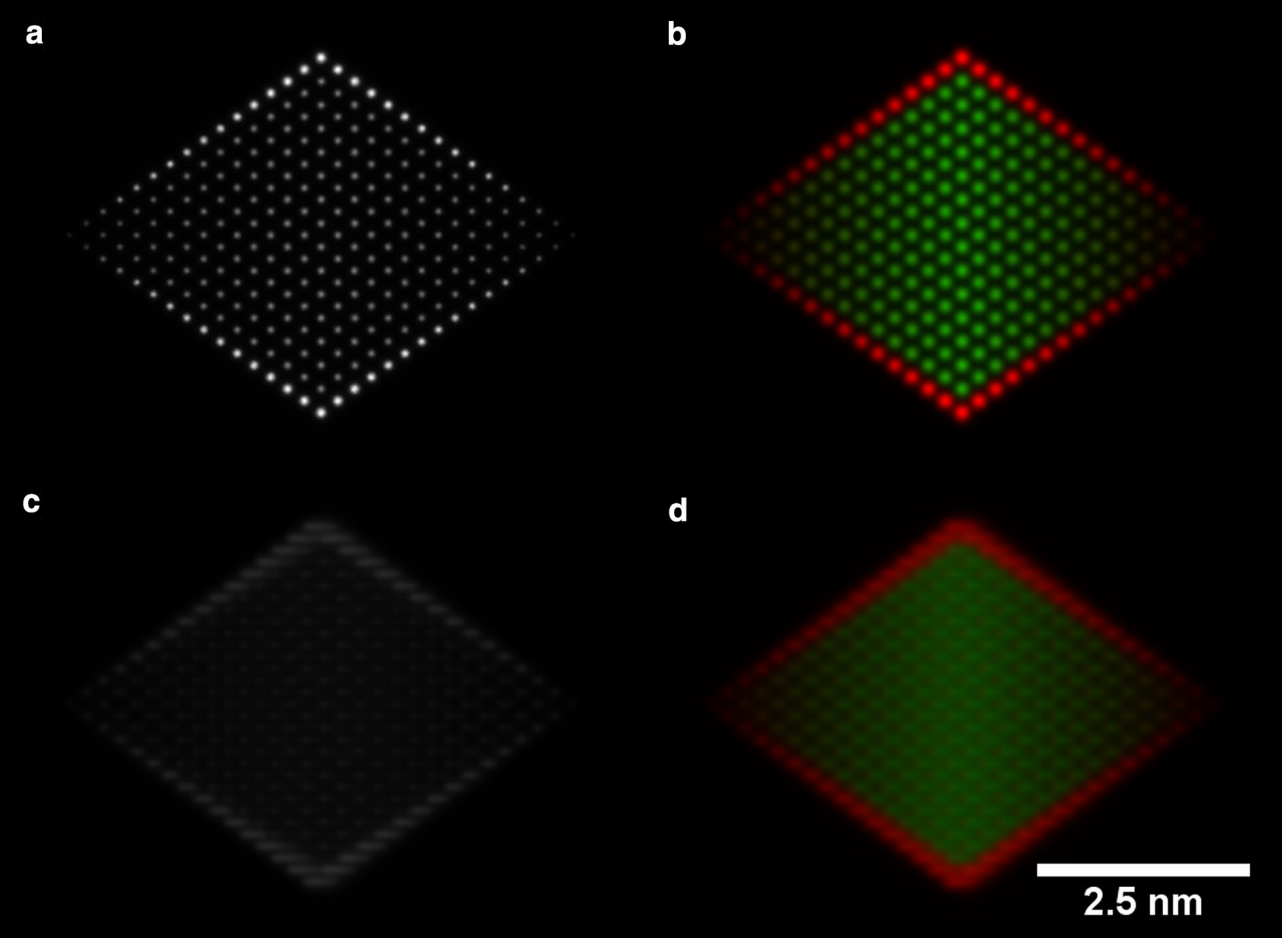
Simulated ADF STEM images and corresponding EDX maps of Pt-shell nanoparticle structure (1), viewed **a**, **b** along a ⟨110⟩ zone axis and **c**, **d** tilted by 5° from the ⟨110⟩ zone axis towards the ⟨100⟩ zone axis. The intensity in **d** has been doubled to make the figure visible on the same intensity scale as the on-axis map (**b**). The Pt-L signal is shown in red, while the Ni-K signal is shown in green

In contrast, corresponding simulations performed for the ⟨110⟩ orientation (i.e. viewed from the edge of the particle) of the same Pt-shell nanoparticle structure, which are shown in Fig. 4, reveal discrete core–shell contrast. In this orientation, four facets are oriented parallel to the electron beam direction, resulting in a clear change from a Ni-rich to a Pt-rich signal around the edge of the nanoparticle. The contrast in the images reflects the change in composition more closely in the ⟨110⟩ orientation than in the ⟨100⟩ orientation. Nevertheless, regardless of the orientation of the nanoparticle, a small amount of sample tilt away from an exact zone axis results in maps that are closer to simple projected thickness profiles and can be easier to interpret.

**Fig. 5:**
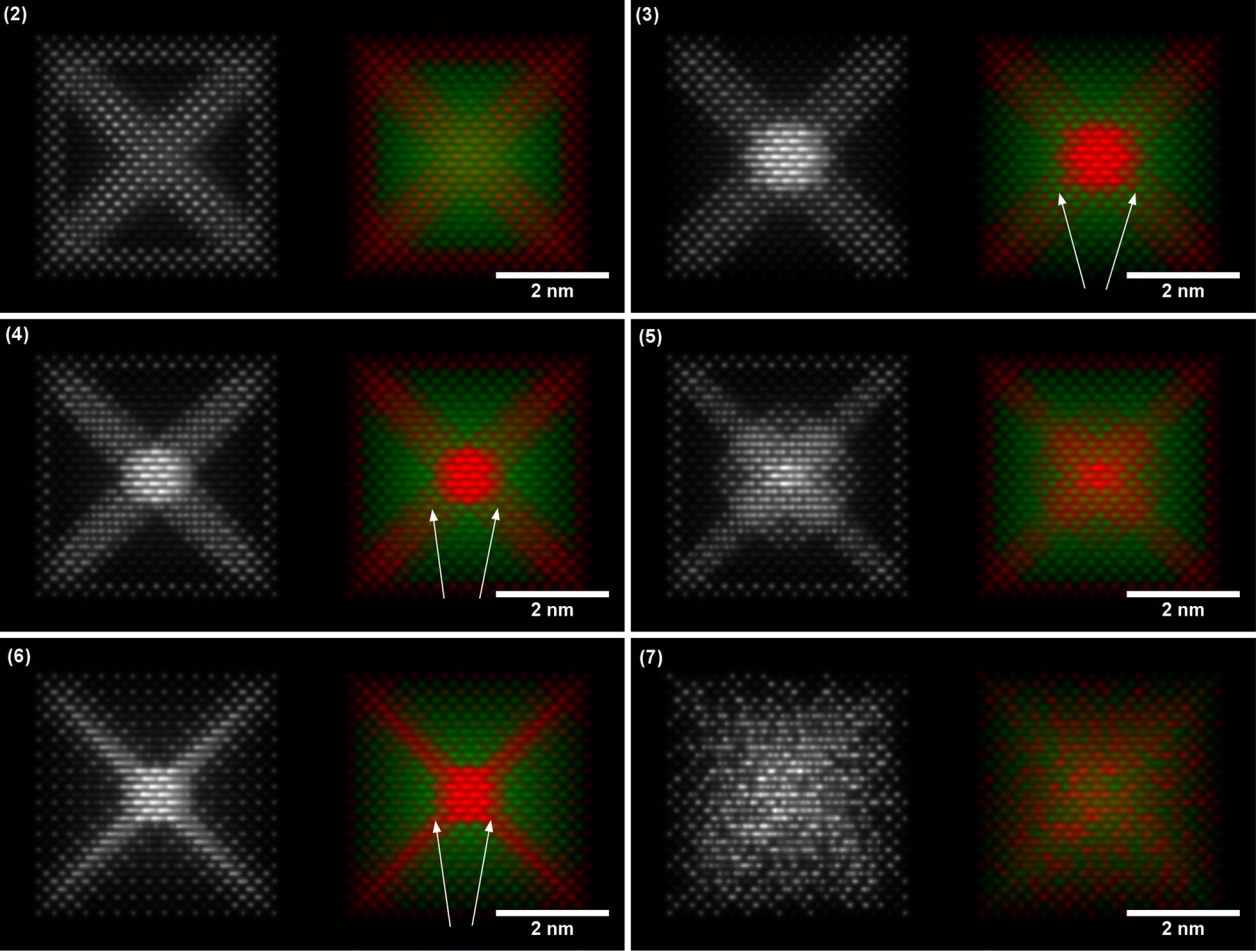
Simulated ADF STEM images and corresponding EDX maps of 6 nanoparticle structures tilted by 5° from the ⟨100⟩ zone axis towards the ⟨111⟩ zone axis: (2) Pt edges, (3) Pt hexapod, (4) Pt hexapod and edges, (5) Pt hexapod, edges and core, (6) Ni facets and (7) alloy. The Pt-L signal is shown in red, while the Ni-K signal is shown in green. The white arrows highlight regions in the EDX maps where the Pt hexapod structure (3) begins to deviate from the Ni facets structure (6)

**Fig. 6:**
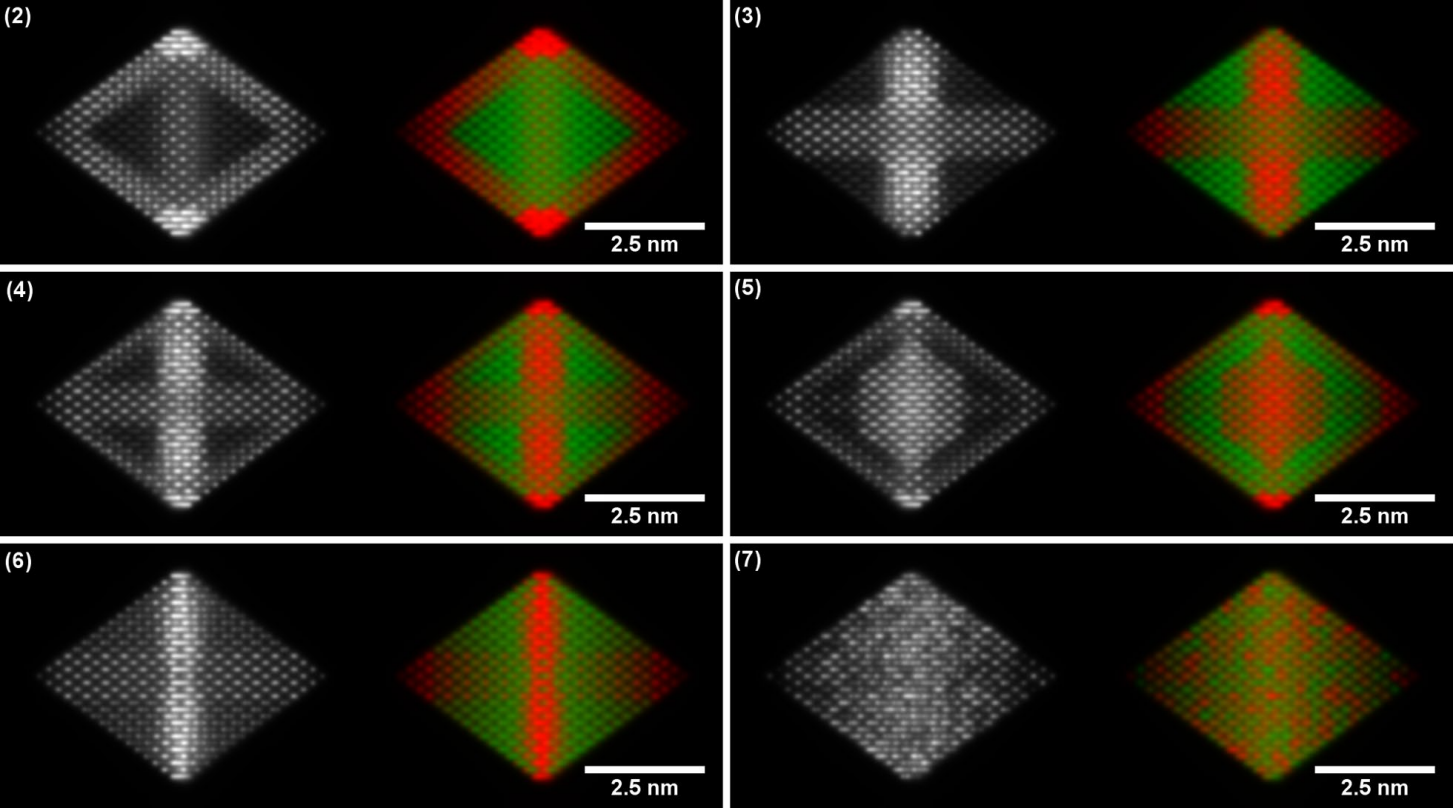
Simulated ADF images and corresponding EDX maps of six nanoparticle structures tilted by 5° from the ⟨110⟩ zone axis towards the ⟨111⟩ zone axis: (2) Pt edges, (3) Pt hexapod, (4) Pt hexapod and edges, (5) Pt hexapod, edges and core, (6) Ni facets and (7) alloy. The Pt-L signal is shown in red, while the Ni-K signal is shown in green

Although nanoparticles are often imaged close to low-order zone axis orientations, they also often rotate as a result of electron beam irradiation [17]. Therefore, images that are simulated for tilted orientations are likely to be more comparable to experimental datasets. Figures 5 and 6 show simulated ADF STEM images and EDX maps for the remaining structures viewed close to ⟨100⟩ and ⟨110⟩ orientations, respectively. These simulations represent EDX maps, in which the projected thickness is the dominating factor that determines the signal, rather than electron channelling. For completeness, Additional file 1: Figures S1–S3 contains simulations performed for an on-axis orientation, as well as an additional tilted orientation from the ⟨110⟩ zone axis, to show that the direction of tilt from the zone axis has little effect for small sample tilt angles.

Close inspection of the images shown in Figs. 5 and 6 can be used to compare the different structures. The alloy nanoparticle (number (6) in Table 1) is completely distinguishable from all of the other structures in both orientations, although even for a truly random alloy (as demonstrated here) there are short-range (column-to-column) fluctuations in both the ADF STEM and the EDX signal. Such variations may not necessarily be visible in experimental datasets, as EDX maps are often acquired at lower spatial resolution to compensate for poor numbers of counts. The Pt-shell structure (number (1) in Table 1) is also clearly distinguishable. However, the other structures are only distinguishable as a result of subtle variations in either the ADF images or the EDX maps. In particular, the Pt hexapod structures (numbers (3) and (4) in Table 1) are difficult to distinguish from the Ni Facets structure (number (6) in Table 1) along the ⟨110⟩ orientation, although they are differentiable in the ⟨100⟩ orientation because the Pt cross shape then begins to disappear in the thicker regions of the nanoparticle when the Ni signal begins to dominate (see arrow markers in Fig. 5(3), (4) and (6)).

**Fig. 7:**
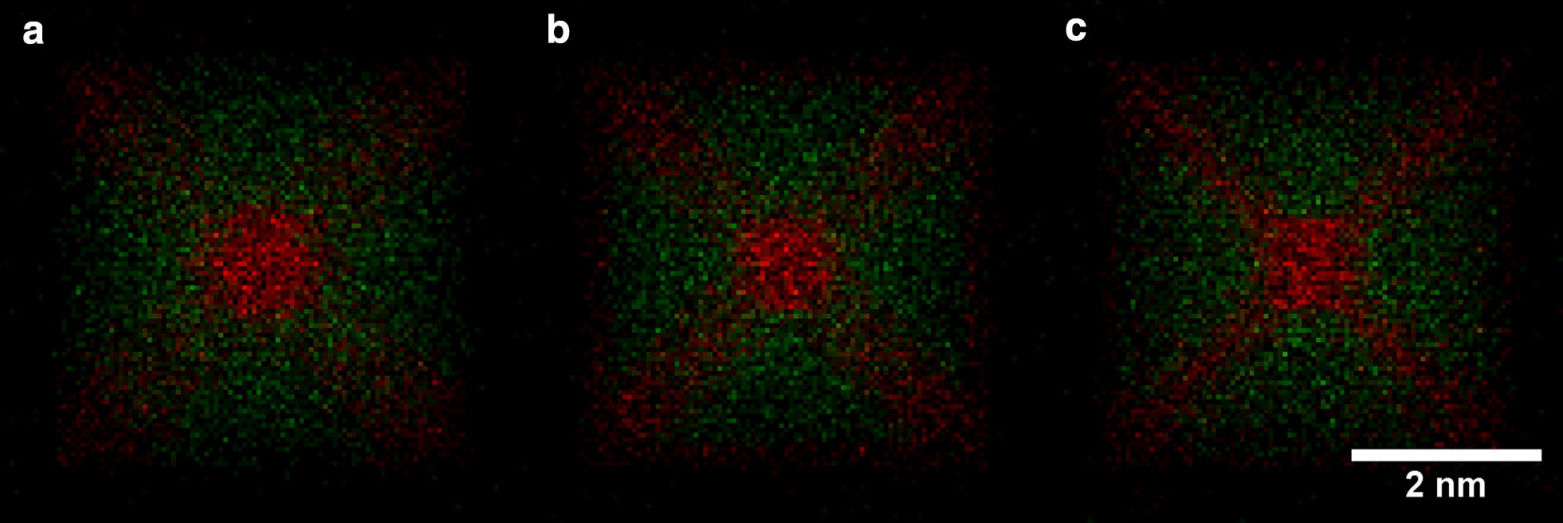
Simulated ‘realistic’ EDX maps, which include the level of Poisson noise that would be expected experimentally for a pixel dwell time of 24.8 ms, pixel width of 50 pm and a total acquisition time of 10 min for the structures: **a** Pt hexapod (3), **b** Pt hexapod and edges (4) and **c** Ni facets (6). The Pt-L signal is shown in red, while the Ni-K signal is shown in green

**Fig. 8:**
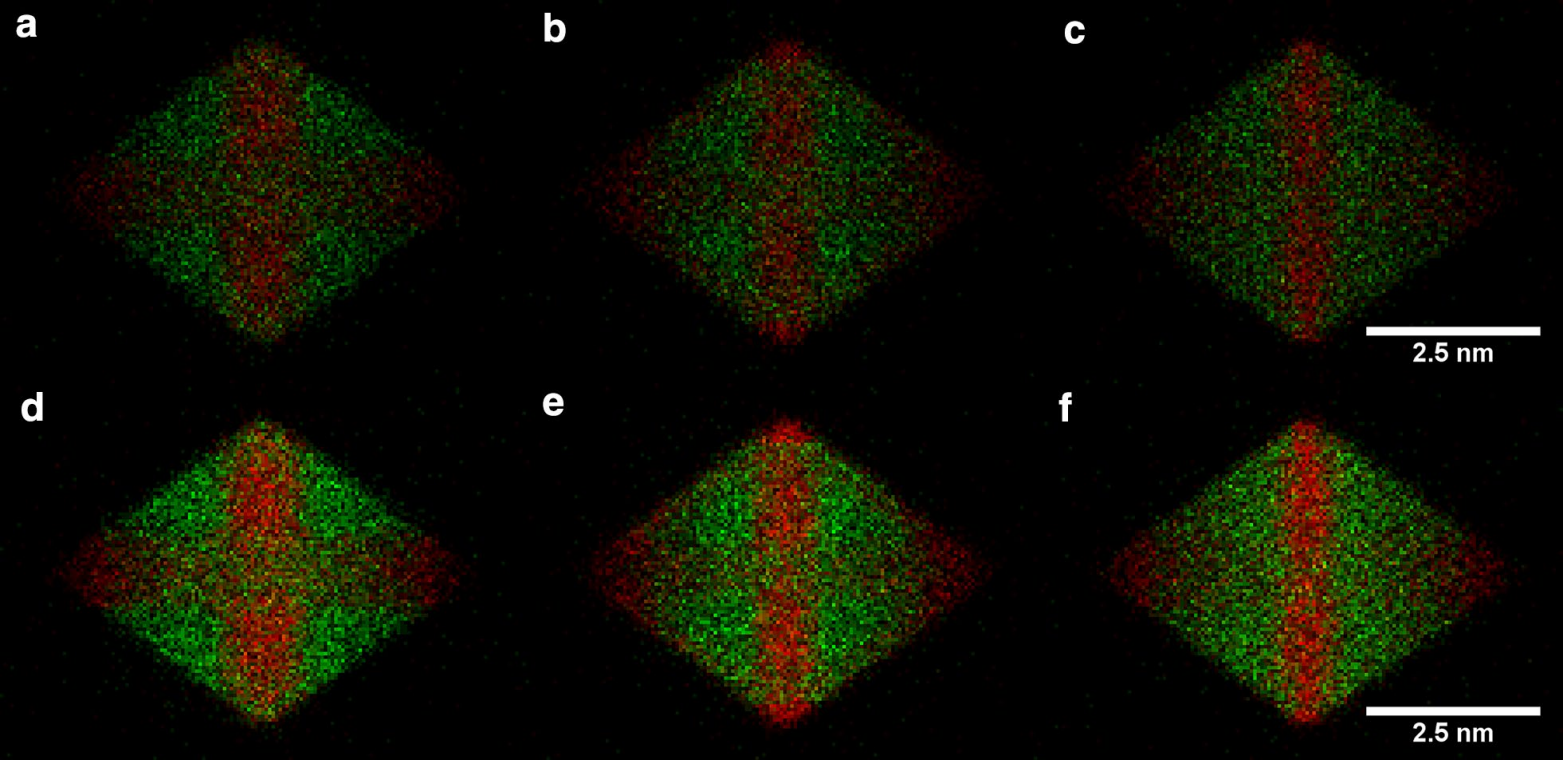
Simulated ‘realistic’ EDX maps, which include the level of Poisson noise that would be expected experimentally for a pixel width of 50 pm and a pixel dwell time of either **a**–**c** 24.8 ms, corresponding to a total acquisition time of 6 min 45 s, or **d**–**f** 49.6 ms, corresponding to a total acquisition time of 13.5 min. The structures are **a**, **d** Pt hexapod (3); **b**, **e** Pt hexapod and edges (4); **c**, **f** Ni facets (6). The Pt-L signal is shown in red, while the Ni-K signal is shown in green

The ⟨100⟩ zone axis does not provide sufficient information to easily distinguish between the structures without (number (3) in Table 1) and with (number (4) in Table 1) Pt decoration along the nanoparticle edges from the EDX maps alone. This point is best illustrated in Fig. 5(3) and (4). The counts are likely to be so low at the edge of the nanoparticle, where its thickness is small, that a distinction between structures (3) and (4) becomes difficult. Figure 7 shows a comparison between structures (3), (4) and (6) once the EDX maps have been converted to absolute counts for a 24.8 ms dwell time per pixel (equivalent to a 10 min total acquisition time for a 110 × 110 pixel map) and after adding Poisson noise. Pt counts are visible at the edge of the particle for structures (4) and (6) (Fig. 7b, c, respectively), but the counts are close to the noise level. A small amount of specimen drift or microscope defocus could easily make this signal less visible. Further simulations for a range of different dwell times are shown in Additional file 1: Figures S4. If the system is known to be binary, then the distinction between the different nanoparticle structures may be more easily made from ADF images (Fig. 5(3) and (4)), in which the higher atomic number of Pt results in an increase in intensity at the nanoparticle edge. The Pt edges structure (number (2) in Table 1) and Pt hexapod and edges structure (number (3) in Table 1) are only discernible in the simulated images as a result of the increased thickness of the Pt decoration (to maintain the same composition). If the edge decoration is similar, then the ⟨110⟩ orientation is better suited for distinguishing between the two structures, as the hexapod no longer overlaps with the Pt edges in projection, as it does in the ⟨100⟩ orientation. This conclusion is confirmed by Fig. 8a, and b, in which the top and bottom corners of the octahedron are brighter for the Pt hexapod and edges structure (3). However, in the ⟨110⟩ orientation, structures (3) and (6) become harder to distinguish, as shown in Fig. 8c. Finally, the structure with a Pt core (5) is much more readily visible in the ⟨110⟩ orientation than in the ⟨100⟩ orientation.

**Fig. 9:**
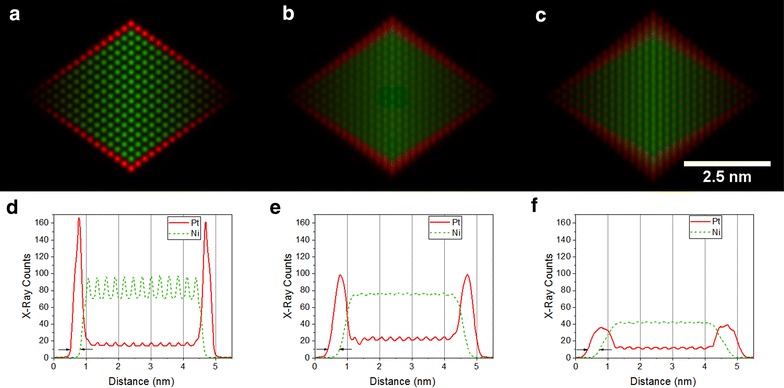
Simulated EDX maps of Pt monolayer nanoparticles viewed **a** along a ⟨110⟩ zone axis, **b** tilted by 5° away from a ⟨110⟩ zone axis and **c** tilted by 10° away from a ⟨110⟩ zone axis. The intensity in **b** and **c** has been doubled to make the figures visible on the same intensity scale as the on-axis map (**a**). Corresponding line profiles taken vertically through the centre of the nanoparticle are shown in **d**, **e** and **f**, assuming a dwell time of 1 s per point and a pixel width of 15 pm. The Pt-L signal is shown in red, while the Ni-K signal is shown in green. The distance used here to measure the shell thickness is highlighted by the black arrows

Although ⟨100⟩ and ⟨110⟩ are the most common zone axis orientations at which nanoparticles are studied in the TEM, nanoparticles may also be oriented away from low-order zone axes. This possibility may have an influence on the measurement of the shell thickness of a core–shell nanoparticle (see Fig. 9). With increasing tilt angle from a zone axis, the shell is expected to look qualitatively thicker in EDX maps, as the atomic columns become less parallel to the incident electron beam direction. EDX line profiles rather than maps are sometimes used to estimate core–shell thicknesses, in part because this approach requires a significantly shorter total acquisition time. Line profiles extracted from each of the simulated EDX maps (Fig. 9d–f) demonstrate how the shell thickness can, in the present case, still be determined despite the presence of sample tilt. The widths of the Pt peaks at the edges of the nanoparticle increase with sample tilt angle. This is because the column of atoms causing this peak becomes less parallel to the electron beam direction. However, the distance between where the signals fall to zero remains approximately constant with sample tilt angle (arrowed in Fig. 9). The measured shell thickness varies by less than 0.1 nm. From a geometric point of view, the measured shell thickness in projection of a uniform shell with thickness *a* varies as $\frac{a}{\cos \theta }$. For small tilt angles, cos*θ* is close to unity, and the measured thickness is very close to the true thickness. Obviously, if the shell is non-uniform, then a greater orientation-dependent variation may of course be present.

**Fig. 10:**
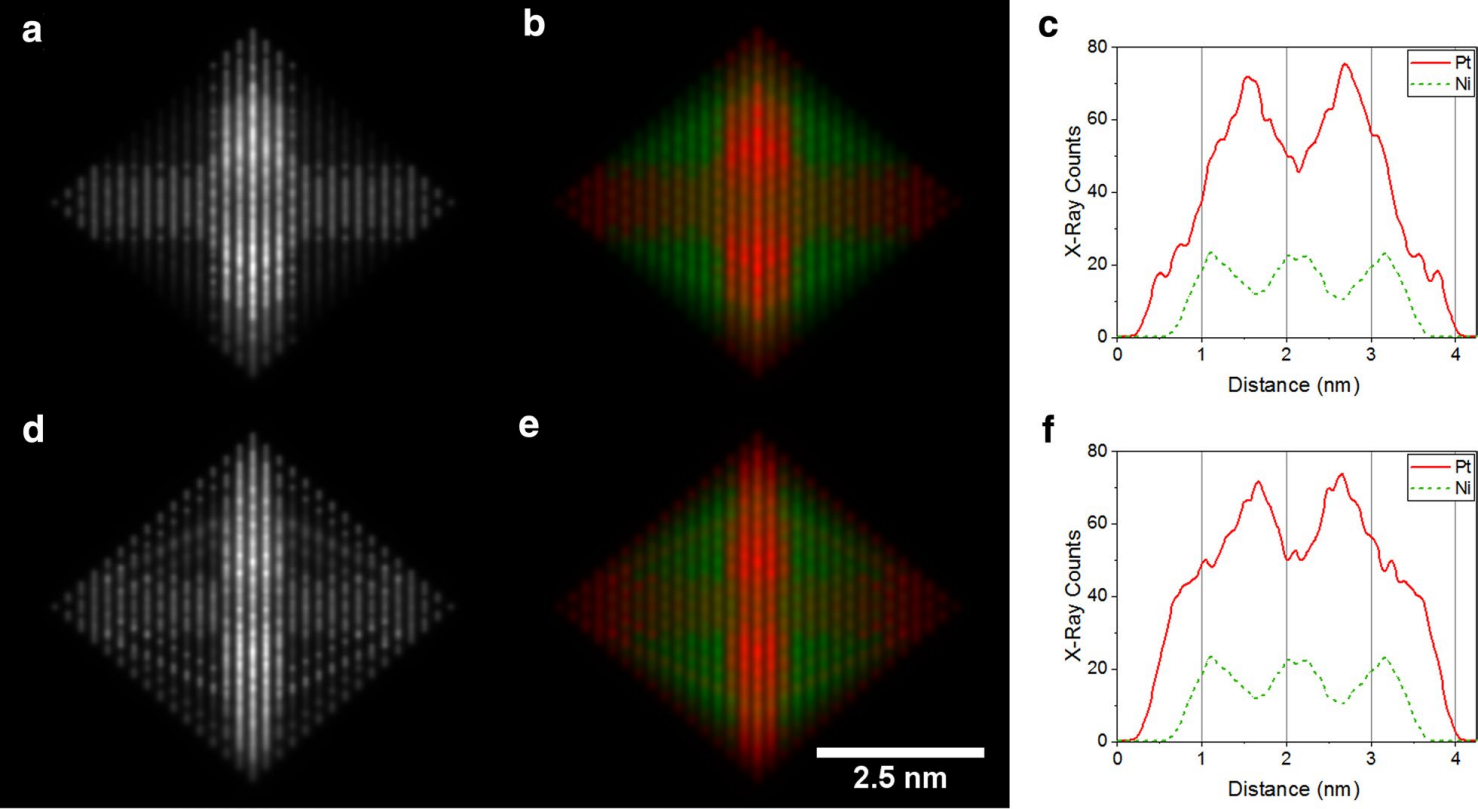
Simulated ADF STEM images and corresponding EDX maps of **a**, **b** Pt hexapod structure (3) and **d**, **e** Pt hexapod + edges structure (4), both tilted by 15° from a ⟨110⟩ zone axis towards the ⟨100⟩ zone-axis. The Pt-L signal is shown in red, while the Ni-K signal is shown in green. Corresponding line profiles taken vertically through the centre of the nanoparticle are shown in **c** and **f**, assuming a dwell time of 1 s per point and a pixel width of 15 pm

The use of larger sample tilt angles could also help with the distinction between nanoparticle structures (3) and (4), as shown in Fig. 10. A simulated ADF STEM image for a tilt angle of 15° from a ⟨110⟩ zone axis (towards ⟨111⟩) reveals the Pt edge decoration as a triangular pattern in Fig. 10d, in contrast to result obtained for the hexapod structure (3) (Fig. 10a). There is also a subtle difference between the EDX maps. For the hexapod structure (3), the Pt band no longer stretches across the entire centre of the nanoparticle (Fig. 10b), whereas for the hexapod and edges structure (4) the Pt appears to be more continuous (Fig. 10e), as well as broader in the line profile (Fig. 10f), although the differences in the ADF image are more distinct.

Because no energy relaxation was carried out on the nanoparticles, the lattice parameter is unchanged from the input value. Small changes in lattice parameter will affect how electrons channel down atomic columns and therefore the resulting intensity. This is another argument for minimising channelling through the use of small sample tilt angles. In the future, lattice strain could be incorporated in simulations using an iterative approach, involving an initial comparison with simulations to obtain a first estimate of the composition, followed by comparison with a new model that incorporates relaxation of the structure.

## Conclusions

In summary, we have presented seven model structures of PtNi octahedral nanoparticles and simulated a library of ADF STEM images and EDX maps for a variety of nanoparticle orientations. This library of simulations highlights the differences between images of the structures generated for different specimen tilt angles. For example, Pt hexapod structures (3) and (4) can be distinguished from a structure that contains continuous Pt planes (6) more readily at a ⟨100⟩ orientation that at a ⟨110⟩ orientation. In contrast, the presence of Pt edge decoration is much more readily visible at a ⟨110⟩ orientation than at a ⟨100⟩ orientation and may be better distinguished when the sample is tilted further away (e.g. 15°) from the ⟨110⟩ zone axis. Although strain was not included in the present study, it could be incorporated into an iterative process once an initial estimate for the composition is known. Such simulations may only need to be carried out once for a given nanoparticle system and provide valuable insight into the 3D structures of highly symmetrical nanoparticles from single 2D maps. An important advantage of such a simulation library is that it saves time at the electron microscope, which may be limited. It can also be used to pre-determine the best parameters to use experimentally for differentiating between two similar structures.

## Additional file

**Additional file 1. Figure S1:** Simulated ADF STEM images and EDX maps of structures (2)-(6) viewed down the <110> zone-axis. **Figure S2**: Simulated ADF STEM images and EDX maps of structures (2)-(6) viewed 5° from the <110> zone-axis towards the <100> zone-axis. **Figure S3**: Simulated ADF STEM images and EDX maps of structures (2)-(6) viewed down the <100> zone-axis. **Figure S4**: Simulated EDX maps from structures (3), (4) and (6) viewed close to the <100> orientation with realistic noise for a selection of dwell times.

## Abbreviations

STEM: scanning transmission electron microscopy
EDX: energy dispersive X-ray
HRTEM: high-resolution transmission electron microscopy
ADF: annular dark-field
ORR: oxygen reduction reaction
