# Supplementary material for: Differentiating the structure of PtNi octahedral nanoparticles through combined ADF–EDX simulations
Source: Adv Struct Chem Imaging. 2018 Feb 20;4(1):2. doi: 10.1186/s40679-018-0053-x (PMC5820384; doi:10.1186/s40679-018-0053-x)
Supplement: Supplementary file 1 — Additional file 1. Figure S1: Simulated ADF STEM images and EDX maps of structures (2)-(6) viewed down the <110> zone-axis. Figure S2: Simulated ADF STEM images and EDX maps of structures (2)-(6) viewed 5° from the <110> zone-axis towards the <100> zone-axis. Figure S3: Simulated ADF STEM images and EDX maps of structures (2)-(6) viewed down the <100> zone-axis. Figure S4: Simulated EDX maps from structures (3), (4) and (6) viewed close to the <100> orientation with realistic noise for a selection of dwell times. [file 40679_2018_53_MOESM1_ESM.pdf]

Differentiating the structure of PtNi octahedral nanoparticles through combined ADF–EDX simulations - Supplementary Information

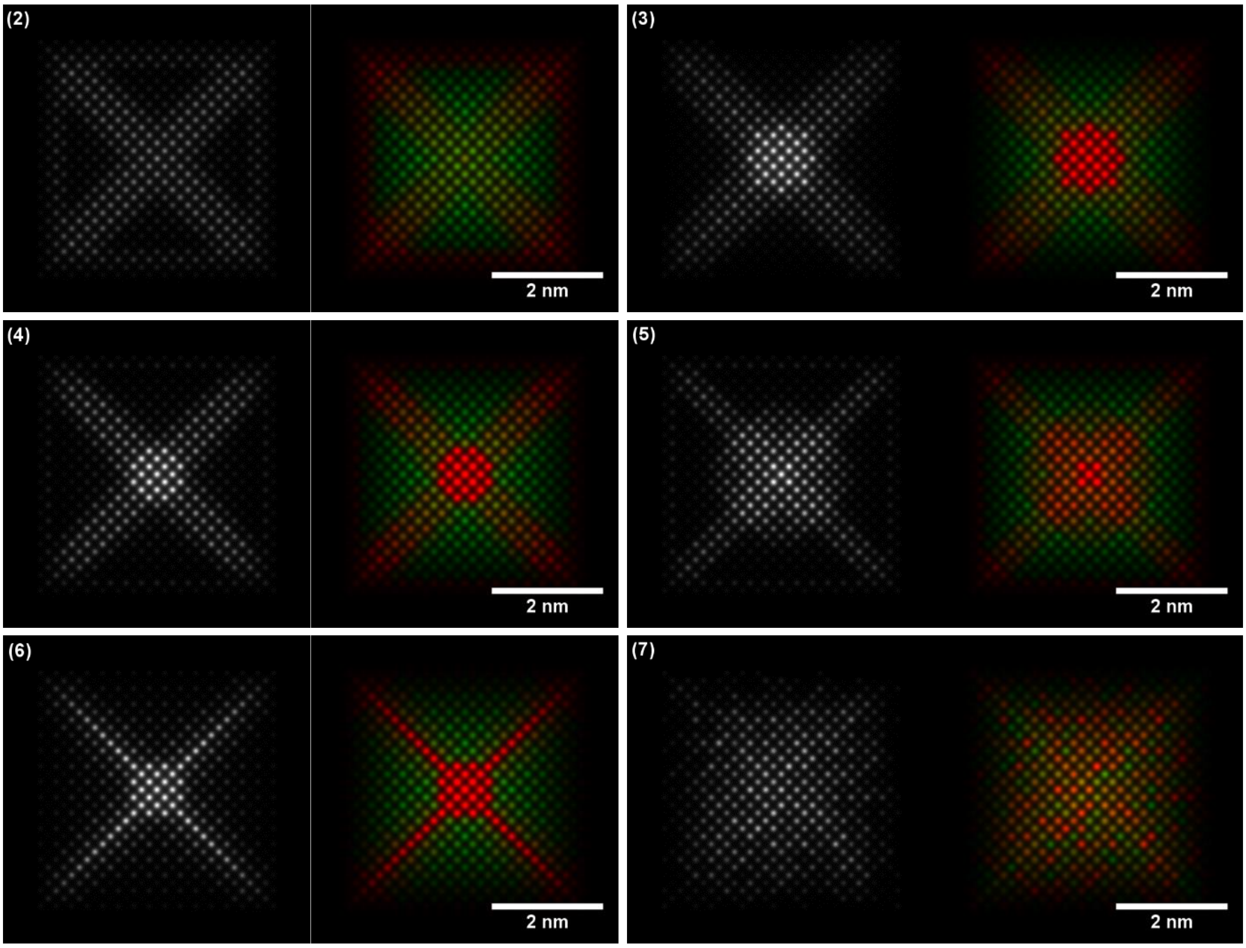

Figure S1: Simulated ADF STEM images and EDX maps of 6 different structures viewed down the <100> zone-axis: (2) Pt edges, (3) Pt hexapod, (4) Pt hexapod and edges, (5) Pt hexapod, edges and core, (6) Ni facets and (7) Alloy. Corresponding EDX maps for the same orientations are also shown. The Pt-L signal is plotted in red, while the Ni-K signal is plotted in green.

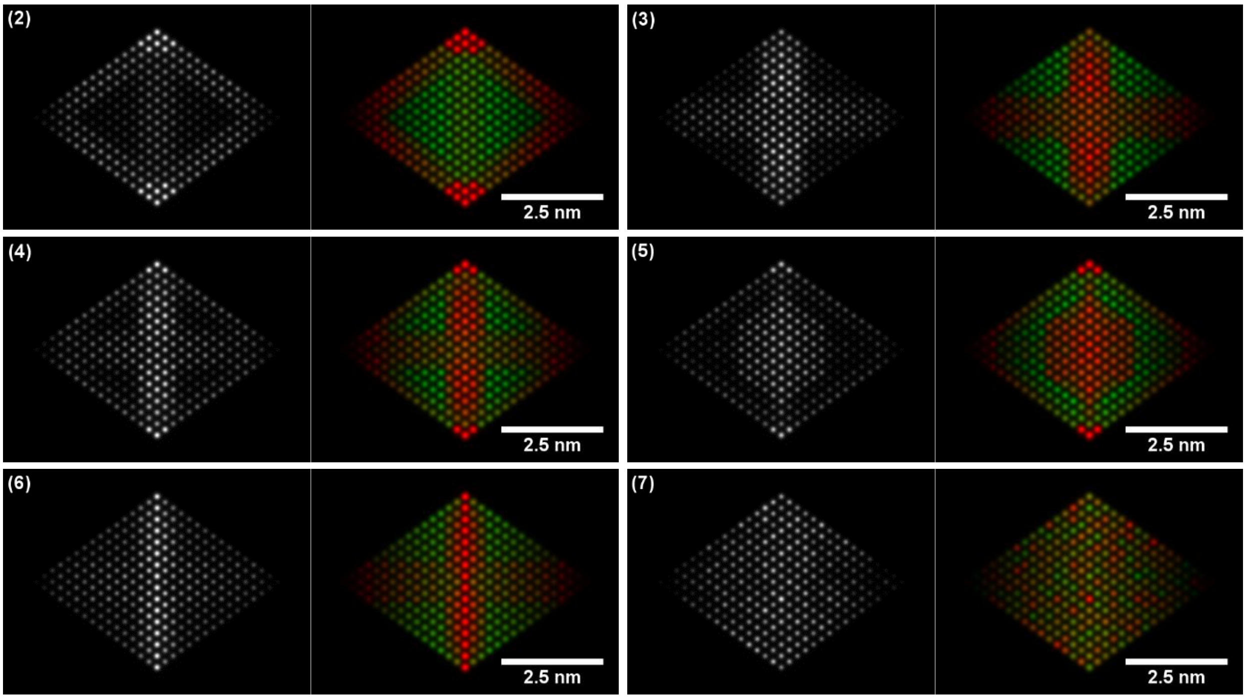

Figure S2: Simulated ADF STEM images and EDX maps of 6 different structures viewed down the <110> zone-axis: (2) Pt edges, (3) Pt hexapod, (4) Pt hexapod and edges, (5) Pt hexapod, edges and core, (6) Ni facets and (7) Alloy. Corresponding EDX maps for the same orientations are also shown. The Pt-L signal is plotted in red, while the Ni-K signal is plotted in green.

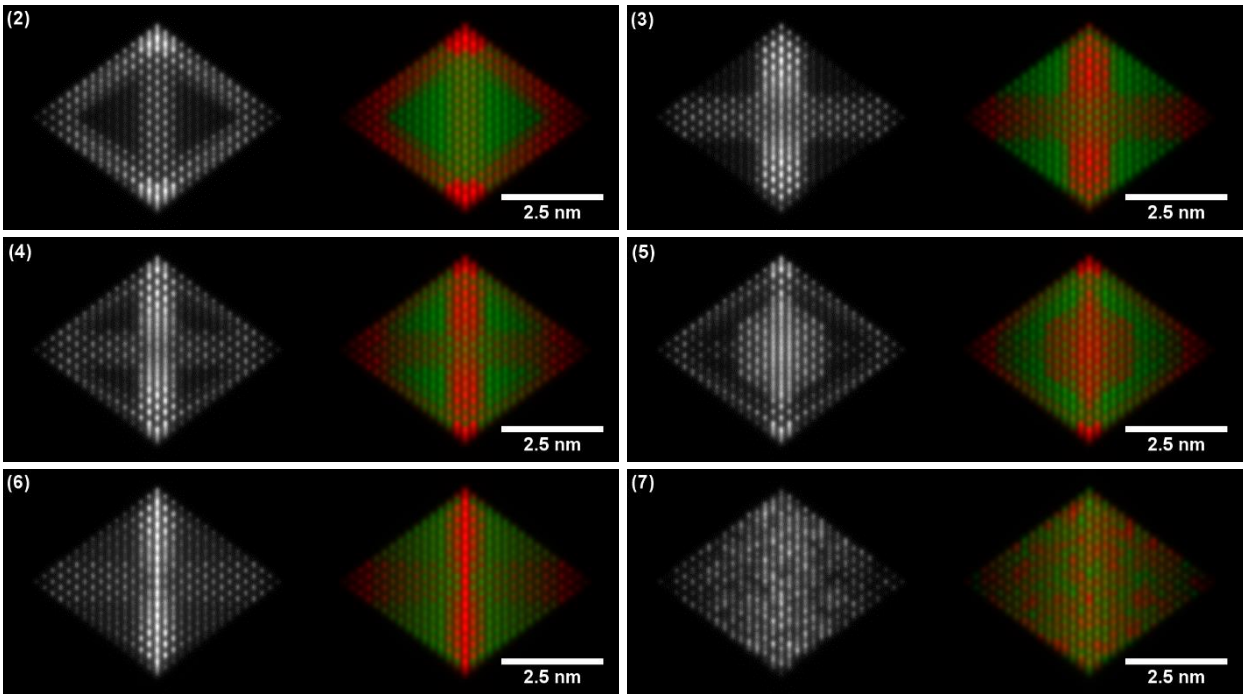

Figure S3: Simulated ADF images and EDX maps of 6 different structures viewed 5° from the <110> zone-axis towards the <100> zone-axis: (2) Pt edges, (3) Pt hexapod, (4) Pt hexapod and edges, (5) Pt hexapod, edges and core, (6) Ni facets and (7) Alloy. Corresponding EDX maps for the same orientations are also shown. The Pt-L signal is plotted in red, while the Ni-K signal is plotted in green.

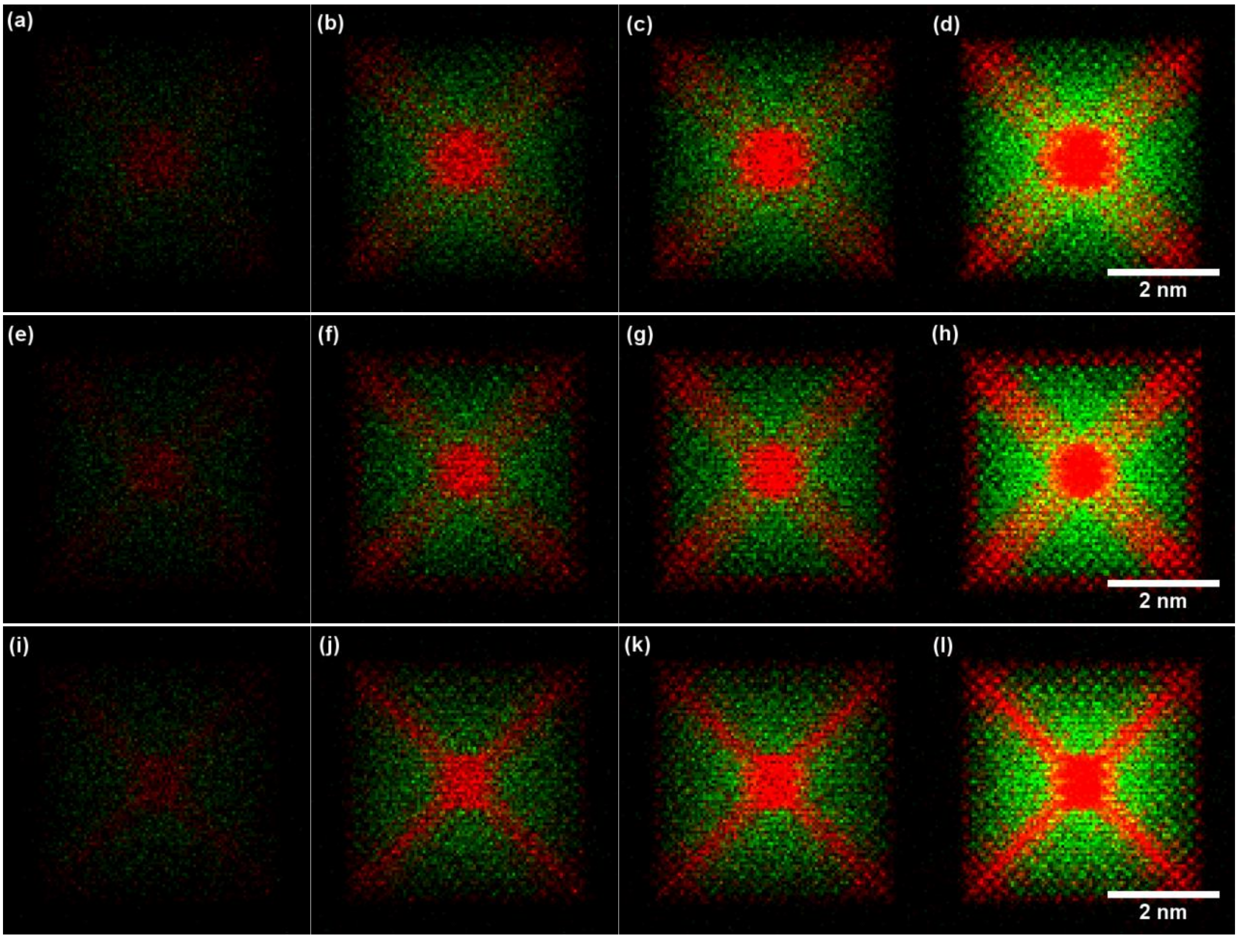

Figure S4: (a-d) Simulated "realistic" EDX maps, including Poisson noise, for Pt hexapod structure (3) for dwell times of (a) 12.4, (b) 49.6, (c) 74.4 and (d) 148.8 ms, corresponding to total acquisition times of 5, 20, 30 and 60 minutes, respectively. (e-h) Corresponding simulated EDX maps for the same dwell times for Pt hexapod and edges structure (4). (i-l) Corresponding simulated EDX maps for Ni facets structure (6). The Pt-L signal is shown in red, while the Ni-K signal is shown in green. The pixel width in these images is 50pm. All images are presented on the same absolute scale, this results in the some saturation of figures (d), (h) and (l) in order to make figures (a), (e) and (i) visible.
